# The tumor and plasma cytokine profiles of renal cell carcinoma patients

**DOI:** 10.1038/s41598-022-17592-3

**Published:** 2022-08-04

**Authors:** Moon Hee Lee, Essi Laajala, Anna Kreutzman, Petrus Järvinen, Harry Nísen, Tuomas Mirtti, Maija Hollmén, Satu Mustjoki

**Affiliations:** 1 Hematology Research Unit Helsinki, Department of Clinical Chemistry and Hematology, University of Helsinki and Helsinki University Hospital Comprehensive Cancer Center, Haartmaninkatu 8, N00290 Helsinki, Finland; 2 Translational Immunology Research Program, University of Helsinki, Helsinki, Finland; 3 Abdominal Center, Urology, University of Helsinki and Helsinki University Hospital, Helsinki, Finland; 4 Department of Pathology, University of Helsinki and Helsinki University Hospital, Helsinki, Finland; 5 Research Program in Systems Oncology, University of Helsinki, Helsinki, Finland; 6 Medicity Research Laboratory, University of Turku, Turku, Finland; 7 iCAN Digital Precision Cancer Medicine Flagship, Helsinki, Finland

**Keywords:** Cancer microenvironment, Tumour biomarkers, Tumour immunology, Urological cancer, Renal cancer, NK cells, T cells

## Abstract

Renal cell carcinoma (RCC) accounts for 90% of all renal cancers and is considered highly immunogenic. Although many studies have reported the circulating peripheral cytokine profiles, the signatures between the tumor tissue and matching healthy adjacent renal tissue counterparts have not been explored. We aimed to comprehensively investigate the cytokine landscape of RCC tumors and its correlation between the amount and phenotype of the tumor infiltrating lymphocytes (TILs). We analyzed the secretion of 42 cytokines from the tumor (n = 46), adjacent healthy kidney tissues (n = 23) and matching plasma samples (n = 33) with a Luminex-based assay. We further explored the differences between the tissue types, as well as correlated the findings with clinical data and detailed immunophenotyping of the TILs. Using an unsupervised clustering approach, we observed distinct differences in the cytokine profiles between the tumor and adjacent renal tissue samples. The tumor samples clustered into three distinct profiles based on the cytokine expressions: high (52.2% of the tumors), intermediate (26.1%), and low (21.7%). Most of the tumor cytokines positively correlated with each other, except for IL-8 that showed no correlation with any of the measured cytokine expressions. Furthermore, the quantity of lymphocytes in the tumor samples analyzed with flow cytometry positively correlated with the chemokine-family of cytokines, CXCL10 (IP-10) and CXCL9 (MIG). No significant correlations were found between the tumor and matching plasma cytokines, suggesting that circulating cytokines poorly mirror the tumor cytokine environment. Our study highlights distinct cytokine profiles in the RCC tumor microenvironment and provides insights to potential biomarkers for the treatment of RCC.

## Introduction

Renal cell carcinoma (RCC) is a heterogeneous cancer that accounts for more than 90% of cancers in the kidney, with the clear cell (ccRCC) subtype as the major cause of kidney cancer-related deaths^1^. Although radical nephrectomy remains the gold standard surgical treatment, approximately 30% of patients with ccRCC with localized disease develop metastases^2–5^. As a tumor that is resistant to radiation and chemotherapy, RCC is also known to be a highly angiogenic, vascularized cancer that expresses VEGF and is counterintuitively characterized by heavy lymphocytic infiltration compared to other solid tumors^6,7^.

Cytokines are essential signaling molecules that elicit an immune response. In addition to regulating host responses to infections, cytokines are also involved in inflammation and dysregulation of the immune system in cancer, diabetes, and viral infections^8,9^. In many solid tumors including RCC, tumor cells acquire various cytokines and their corresponding receptors from the surrounding normal stroma to grow, proliferate, and survive^9,10^. Although previous studies have analyzed the circulating cytokines in the blood^11,12^, comparisons between the cytokines released from the tumor and matching adjacent healthy renal tissue have not been fully explored.

Our recent data showed immunological differences between RCC tumors that displayed T and NK cell dominant immune phenotypes^13^. In this study, we aimed to explore the underlying cytokine signatures that are present within the tumor environment, adjacent healthy renal tissue, as well as those in circulation, and in turn, correlate the profiles with the immunophenotype and clinical data.

## Materials and methods

### Patients and study approval

The study included newly diagnosed RCC (n = 46) patients that underwent radical nephrectomy (Supplemental Table S1). The primary tumor (n = 46), adjacent healthy renal tissue (n = 23), and peripheral blood samples (n = 33) were obtained from the patients during the surgical procedures within a four-year time frame.

The study was approved by the Helsinki University Hospital ethical committee (Dnro 115/13/03/02/15) and was conducted in accordance with the Declaration of Helsinki. All patient samples were taken after a signed informed consent.

### Sample preparation and processing

A prospective sample cohort of freshly excised tumor and matching adjacent healthy tissue samples were collected (2016–2020) and stored in MACS® tissue storage solution (Miltenyi Biotec 130-100-008) at 4 °C upon harvest. All samples were processed directly upon arrival at our facilities within minimal transportation time. Each sample was independently dissociated using Miltenyi’s Tumor Dissociation kit protocol (Miltenyi Biotec 130-095-929). A portion of the freshly dissociated cells were viably frozen in 10% FBS-DMSO solution and preserved at − 150 °C for the cytokine assay. Plasma samples were initially separated from the peripheral blood using centrifugation (300 g, 10 min). Isolated plasma samples were further centrifuged (1300 g, 10 min) and stored in cryo vials at − 70 °C until further use.

### Multi-parameter flow cytometry and immunophenotyping

From our previous study, freshly dissociated tumor and adjacent healthy kidney samples were used to examine the immune cell numbers and immunophenotypes, revealing two subgroups of RCC tumors defined by CD3 + T cell and NK cell abundance, as well as various marker expressions^13^. Briefly, the dissociated samples were stained for 15 min with a comprehensive antibody staining panel that included markers for immune checkpoint molecules, chemotaxis, cytotoxicity, and cell migration (CD3, CD4, CD8, CD11b, CD16, CD25, CD27, CD56, CD57, CD161, CTLA-4, CX3CR1, CXCR3, CXCR4, CCR7, CD45RO, ICOS, HLA-DR, LAG-3, PD-1, TCRγδ) as previously described^13^. A total of 50 000 lymphocytes were acquired with the FACS Verse (BD Biosciences) and analyzed with FlowJo (Version 10.0.8rl, Treestar). All antibodies were purchased from BD Biosciences (BD Biosciences, San Diego, CA, USA) unless mentioned otherwise.

### Clinical data

An assessment of 18 clinical parameters (tumor size and weight, TNM staging, WHO/ISUP 2016 tumor grading simplified into two classes: low (G1-G2) and high (G3-G4), presence of necrosis, peri-renal and peri-pelvic fat infiltration, rhabdoid histology, age at relapse) and other medical histories were included (Supplemental Table S1).

### Conditioned media

Conditioned media were obtained from each dissociated tumor (n = 46) and adjacent healthy renal tissue (n = 23) sample. First, 100 000 cells in 150μL/well were seeded in 96-U well culture plates and cultured in tumor cell media (RPMI-1640, 10% FBS, 1% Penicillin/Streptomycin, 2 mM L-Glutamine, 10 mM sodium pyruvate, hydrocortisone sodium succinate (Solu-Cortef) 0.0004 mg/mL) for 24 h at 37 °C, 5% CO_2_. The next day, cells were washed twice with PBS to remove any remaining serum, replaced with unsupplemented RPMI-1640, and were incubated for 24 h at 37 °C, 5% CO_2_. The conditioned media were collected and filtered through a 0.2 μm syringe before use. Details of the conditioned media protocol have previously been described in detail^14,15^.

### Multiplex cytokine assay

The presence of various cytokines such as chemokines, growth factors, and interleukins (pg/mL) from the serum-free conditioned media and 100μL of plasma samples were analyzed using the Bio-Plex Pro™ human cytokine screening panel (Bio-Rad) according to the manufacturer’s instructions.

### Statistical analyses

#### Tumor-healthy comparisons

**Figure 1:**
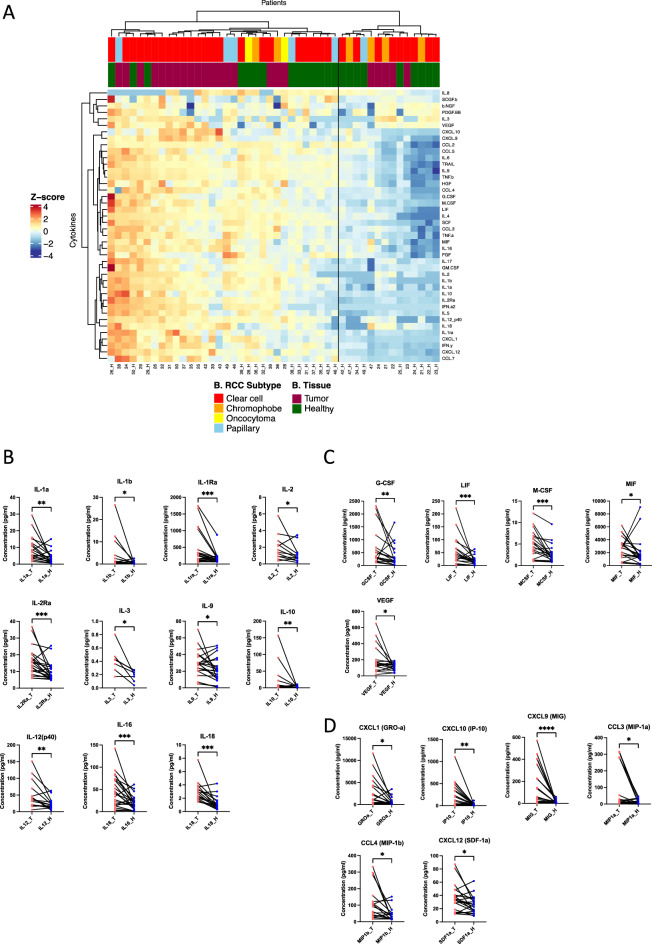
(**A**) Heatmap showing the cytokine profile of 23 matching RCC tumors and healthy adjacent tissue samples using unsupervised clustering. Upper color keys represent clinical parameters (RCC histology and type of tissue sample). Euclidean distance clustering and ward.D2 linkage methods were used from log_2_ transformed data values. Numerical identifiers at the bottom of the heatmap refer to individual patient tumors, where “_H” codes correspond to the healthy tissue counterparts. From the cytokine profiles, patients may be divided into two large clusters – those with high and those with low cytokine expression profiles. Results including all tumor samples and only ccRCC cases are found in Supplementary Fig. S1A,B. (**B**–**D**) Comparison of each cytokine profile between the matching tumor and healthy adjacent renal tissue samples show significant differences, with the tumors generally displaying a higher expression and broader range of cytokines. The cytokines were grouped based on their biological qualities: (**B**) interleukin families, (**C**) growth, inhibiting and stimulating factors, and (**D**) chemokines. All other cytokines are found in Supplemental Fig. S2A–E. Wilcoxon matched-pairs signed rank statistical testing with a 95% confidence level was used. *p < 0.05; **p < 0.01; ***p < 0.001; ****p < 0.000.1

Wilcoxon matched-pairs signed rank test with a 95% confidence level was used for paired tumor-healthy samples (Fig. 1B–D, Supplementary Fig. S2A–E). Non-parametric Mann–Whitney U-test (unpaired, two-tailed) with a 95% confidence level was used to compare two groups of absolute cytokine concentrations (Supplementary Fig. S1C–E). All scatter dot plots show error bars with the median and range as horizontal lines. The statistical analyses were performed using Prism 9 Version 9.2.0 (GraphPad Software Inc.). For all graphs: ns, not significant; *p < 0.05; **p < 0.01; ***p < 0.001; ****p < 0.0001.

#### Heatmap and correlation analyses

**Figure 2:**
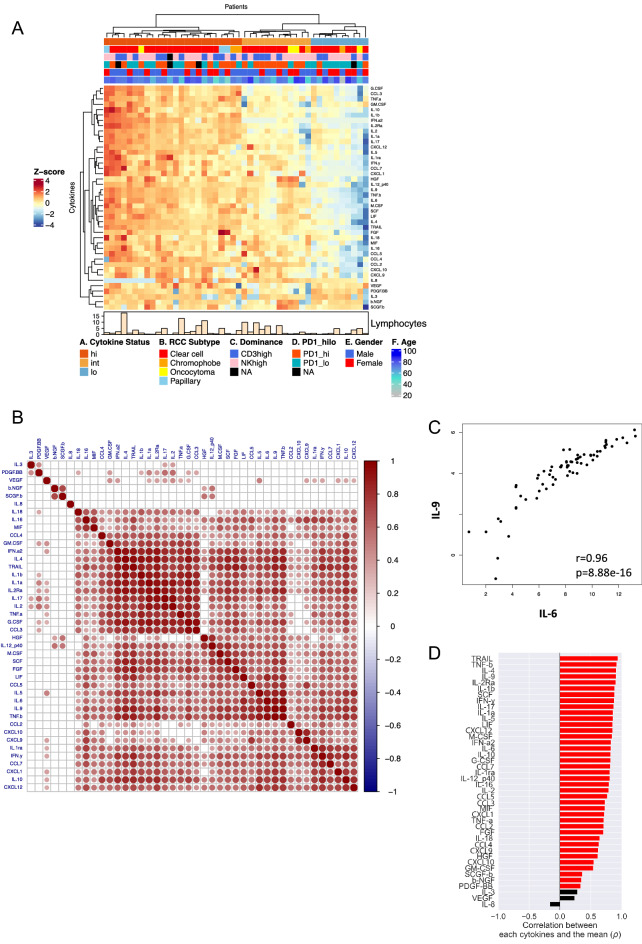
(**A**) Heatmap of RCC tumors expressing a total of 42 cytokines, with clinical parameters such as the RCC subtype, gender, and age. Unsupervised Euclidean distance clustering and ward.D2 linkage methods were used. RCC tumors have a distinct cytokine profile based on three clusters ((A) Cytokine Status): those with high (hi), intermediate (int), and low (lo) cytokine expressions. No marked differences were observed between the clinical parameters. The (C) Dominance and (D) PD1_hilo color keys refer to our previous studies^13^ with RCC tumors that were subgrouped into CD3 + T and NK cell dominant tumors (Supplemental Fig. S3A), or CD4 + and CD8 + T cell PD-1 expression (Supplemental Fig. S3B). NAs refer to samples excluded from the analysis or with missing clinical data. (**B**) Correlation plot using Spearman rank correlation shows strong positive correlations across most of the cytokines expressed in the tumor. However, different expression patterns were observed for a few cytokines (IL-3, PDGF-BB, VEGF, β-NGF, and SCGF-β). IL-8 was the only cytokine that showed no correlation with any of the measured cytokine levels. Only statistically significant cytokines (Bonferroni-Hochberg corrected with a false discovery rate (FDR) < 0.05) are shown. (**C**) Spearman’s correlation plot showing IL-6 and IL-9 as one of the strongest positively correlating cytokines. (**D**) Pearson’s correlation analysis using the python-based tool, CytoMod^18^, shows correlations between each absolute cytokine expression and mean cytokine levels of each tumor sample, revealing significantly positive correlations, except for IL-8 that showed a negative correlation.

Unsupervised heatmap clustering was carried out by using the Euclidean distance and ward.D2 linkage method after log_2_ transforming and Z-score scaling the data. The Z-score scaling was performed for each cytokine (row). Spearman’s correlation and hierarchical clustering with the complete linkage method were used for the correlation plot (Fig. 2), which was created with the R package, corrplot^16^. R version 4.0.4.^17^, RStudio version 1.3.1056 and the Python package CytoMod^18^ were used for the analyses. Benjamini and Hochberg-corrected *p*-values with a false discovery rate (FDR) < 0.05 with were considered significant.

## Results

### RCC tumors display a distinct cytokine profile compared to the healthy renal tissue counterparts

First, we assessed the cytokine landscape of the RCC tumors in comparison to the profiles of the adjacent healthy renal tissue samples. Our cohort included 46 tumor cases, out of which 35 (76.1%) were ccRCC, 4 (8.7%) chromophobe RCC, 3 (6.5%) papillary RCC, and 4 (8.7%) benign oncocytoma cases, confirmed by histopathological analysis (Table 1). From a total of 46 tumor cases, we selected 23 available matching healthy renal tissue samples that were histopathologically confirmed to exclude any tumor cells.

**Table 1:** Sample cohort and patient characteristics.

Patients	All (n = 46)
Tumor	46
Matching adjacent healthy renal tissue	23
Age in years: mean (range)	66 (23–85)
Sex: n (%)	
Male	27 (58.7)
Female	19 (41.3)
Tumor histology: n (%)	
Clear cell	35 (76.1)
Chromophobe	4 (8.7)
Papillary	3 (6.5)
Oncocytoma (benign)	4 (8.7)
WHO/ISUP 2016 tumor grade at diagnosis: n (%)	
I-II	23 (50)
III-IV	19 (41.3)
Unknown	4 (8.7)
Deaths due to metastasis: n (%)	
Yes	2 (4.3)
No	44 (95.7)

Using an unsupervised approach, we analyzed the cytokine profiles of the 23 matching tumor and healthy tissues (Fig. 1A). Our heatmap analysis showed that the samples were divided into two main clusters: samples with high cytokine expressions and those with low expressions. 14 out of 32 samples (43.7%) in the highly expressing group were healthy tissue samples, compared to 9/14 samples (64.3%) in the lowly expressing cytokine group. All the healthy tissue samples belonging to the high cytokine expression cluster had their matching tumor tissues in the same cluster. In the lowly expressing cytokine cluster, five paired healthy-tumor samples belonged to the same group, whereas in four healthy cases, the corresponding tumor samples belonged to the high cytokine expression cluster. When we repeated the analysis for all tumor (n = 46) and healthy samples (n = 23) (Supplementary Fig. S1A), as well as only ccRCC cases (Supplementary Fig. S1B), we similarly observed two main clusters (high and low cytokine expressions). 13/23 (56.5%) healthy tissues belonged to the low cytokine expression cluster when including all samples, whereas the majority of the healthy samples (13/17, 76.5%) belonged to the low expression cluster in the ccRCC cases alone.

Next, we compared in detail the cytokine profiles between the matched tumor and healthy adjacent renal tissues, and discovered significant differences between the samples in 22 out of a total of 42 measured cytokines. Overall, the cytokine levels in the tumor (T) tissues were greater compared to the healthy (H) counterparts (Fig. 1B–D). Similar results were observed when all the tumor and healthy samples were included, except in FGF, where a moderate increase in concentration was observed in the healthy tissues than in the tumor samples (Supplementary Fig. S1C-E). The biggest differences between the matched tumor-healthy samples were detected in the interleukin and growth factor concentrations: IL-1Ra (*p* = 0.0006, median 237.8 pg/mL vs 135.1 pg/mL), IL-2Ra (*p* = 0.0004, median 14.5 pg/mL vs 7.3 pg/mL), IL-16 (*p* = 0.0006, median 40.7 pg/mL vs 15.6 pg/mL), IL-18 (*p* = 0.0001, median 2.8 pg/mL vs 1.0 pg/mL) (Fig. 1B); LIF (*p* = 0.0007, median 38.9 pg/mL vs 15.3 pg/mL) and M-CSF (*p* = 0.0005, median 4.7 pg/mL vs 2.1 pg/mL) (Fig. 1C). Additionally, the levels of the chemokines CXCL9 (MIG, *p* < 0.0001, median 54.3 pg/mL vs 7.0 pg/mL) and CXCL10 (IP-10, *p* = 0.0021, median 92.9 pg/mL vs 16.7 pg/mL) were also increased in the tumor samples compared to the healthy tissues (Fig. 1D). Full comparisons between the tumor and healthy cytokines are found in the Supplemental Fig. S2A–E.

### Intratumoral cytokines highly correlate with each other

To better understand the individual differences in the patient samples, we independently analyzed the cytokine profiles in the tumor samples using the same unsupervised approach and observed three main clusters: tumors with high (hi), intermediate (int), and low (lo) cytokine expressions (Fig. 2A). 24 out of 46 (52.2%) tumor cases belonged to the hi group, whereas 12 (26.1%) and 10 (21.7%) samples belonged to the cytokine int and lo groups, respectively. No differences were found in the clinical parameters such as gender, age, WHO/ISUP 2016 tumor grading, and presence of necrosis between the clusters. Similarly, although tumors were divided into distinct subgroups based on the immune profiles (CD3 + T and NK cell dominant) from our previous study^13^ (Supplemental Fig. S3A), and PD-1 high (PD1_hi) and low (PD1_lo) based on the CD4 + and CD8 + T cell PD-1 expressions (Supplemental Fig. S3B), the subgroups were evenly distributed among the cytokine profiles. The quantity of tumor lymphocytes neither differed between the cytokine high, intermediate, and low clusters (Supplementary Fig. S3C).

Subsequently, using Spearman’s rank correlation and false discovery rate (FDR) < 0.05 across the tumor cytokines, we discovered strong positive correlations across the majority of the expressed cytokines (Fig. 2B), suggesting that tumors expressing high levels of a particular cytokine were likely to exhibit increased expression signatures of the other cytokines as well. One of the highest positive correlations was found between IL-6 and IL-9 (Fig. 2C). Contrarily, IL-3, PDGF-BB, VEGF, β-NGF, and SCGF-β showed positive correlations with only a handful of cytokines, making their expression profiles different from the rest. IL-8 displayed no correlation with any of the measured cytokines and was lowly expressed in a proportion of the samples that belonged to the cytokine hi cluster (Fig. 2A,B,D). Apoptosis-related cytokines such as TRAIL, TNF-β and IFN-γ were among the top cytokines that exhibited the highest correlations, whereas IL-8 displayed a negative correlation between the overall mean tumor cytokine levels (Fig. 2D). None of the tumor cytokines correlated with age, sex, metastasis, and presence of necrosis from all tumor samples (Supplementary Fig. S3D), as well as in only ccRCC cases (Supplementary Fig. S3E). Using the same unsupervised hierarchical clustering methods, we also analyzed the cytokine profiles of the healthy adjacent renal tissues (n = 23). Similarly, the healthy tissue samples clustered into three clusters based on the cytokine expressions (hi, int, and lo) (Supplemental Fig. S4A). As observed with the tumor tissues, most of the cytokines in the healthy tissues positively correlated with each other in their expression profiles, except for IL-8. (Supplemental Fig. S4A,B).

### The expression of CXCL10 and CXCL9 are correlated with tumor lymphocyte abundance

**Figure 3:**
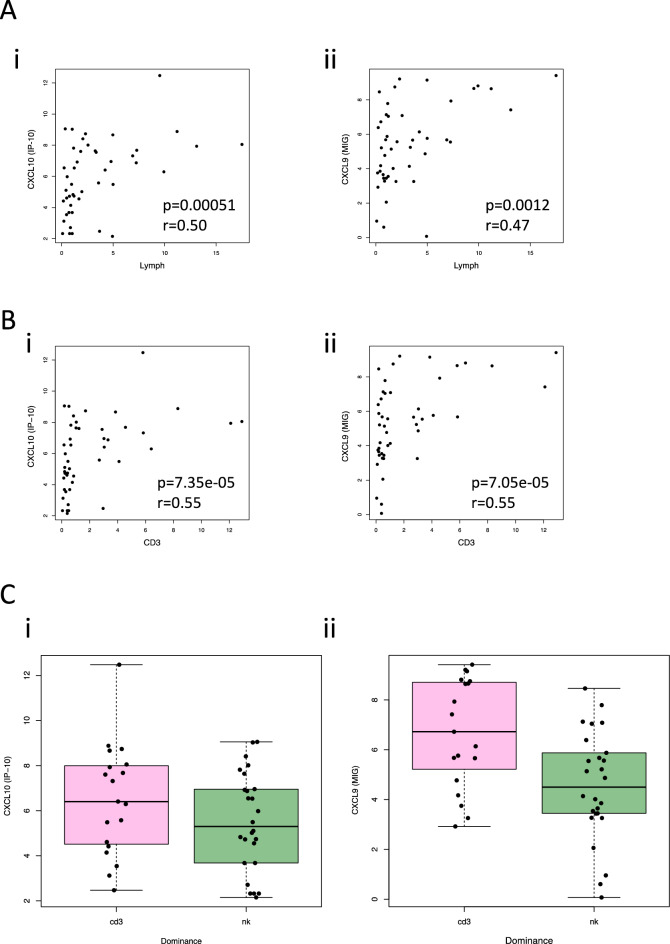
(**A**) Scatterplots showing Spearman’s correlation between the expression of various cytokines (CXCL10 and CXCL9) with the overall quantity of lymphocytes in the tumor samples: (i) CXCL10 vs Lymphocytes; (ii) CXCL9 vs Lymphocytes. Lymph = Lymphocytes. CXCL10 and CXCL9 showed the strongest positive correlations with the abundance of lymphocytes in the tumor. *p* = *p*-value, r = Spearman’s rho. (**B**) Scatterplots showing positive Spearman’s correlations between the expression of the cytokines and CD3 + T cell abundance out of all cells in the tumor: (i) CXCL10 vs CD3, (ii) CXCL9 vs CD3. *p* = *p*-value, r = Spearman’s rho. (**C**) Boxplots comparing the expression of (i) CXCL10 and (ii) CXCL9 with tumor immune cell dominance. CXCL9 was significantly elevated in the CD3 + T cell dominant compared to the NK cell dominant tumors (*p* = 0.0044).

We next sought to investigate whether certain cytokines would impact the immune cell infiltration in the tumor. Although the tumor cytokine profiles did not cluster based on the CD3 + T and NK cell dominant immunophenotypes (Fig. 2A), we analyzed the cytokine profiles in relation to the quantity and phenotype of the TILs. The total amount of TILs in tumor samples did not significantly differ between the hi, int, and lo cytokine tumor groups (Supplemental Fig. S3C). However, when individual cytokines were examined, CXCL10 and CXCL9 expressions significantly correlated with lymphocyte abundance (Fig. 3Ai,ii, Supplementary Fig. S3D, E). We further analyzed the quantity of CD3 + T and NK cells out of all cells in the tumor samples and observed that CXCL10, and CXCL9 levels were significantly correlated with the number of tumor infiltrating CD3 + T cells (Fig. 3Bi,ii); CXCL10 levels also correlated with the number of tumor infiltrating NK cells (Supplementary Fig. 5i,ii). Similar observations were made when only the ccRCC cases were included; significant positive correlations were observed for both CXCL10 and CXCL9 with the tumor lymphocytes and CD3 + T cells, respectively, but not with the NK cells (Supplementary S5Bi-vi). Although elevated CXCL10 expression was increased in the CD3 + T cell dominant tumors compared to the NK dominant counterparts in all the RCC and ccRCC cases, no statistical differences were observed (Fig. 3Ci and Supplementary Fig. S5Ci, respectively). However, CXCL9 levels were significantly higher in the CD3 + T cell dominant tumors compared to NK cell dominant tumors in all (*p* = 0.0044) as well as the ccRCC cases (*p* = 0.03) (Fig. 3Cii and Supplementary Fig. S5Cii, respectively).

### Tumor cytokine profiles are markedly different from circulating plasma cytokines

**Figure 4:**
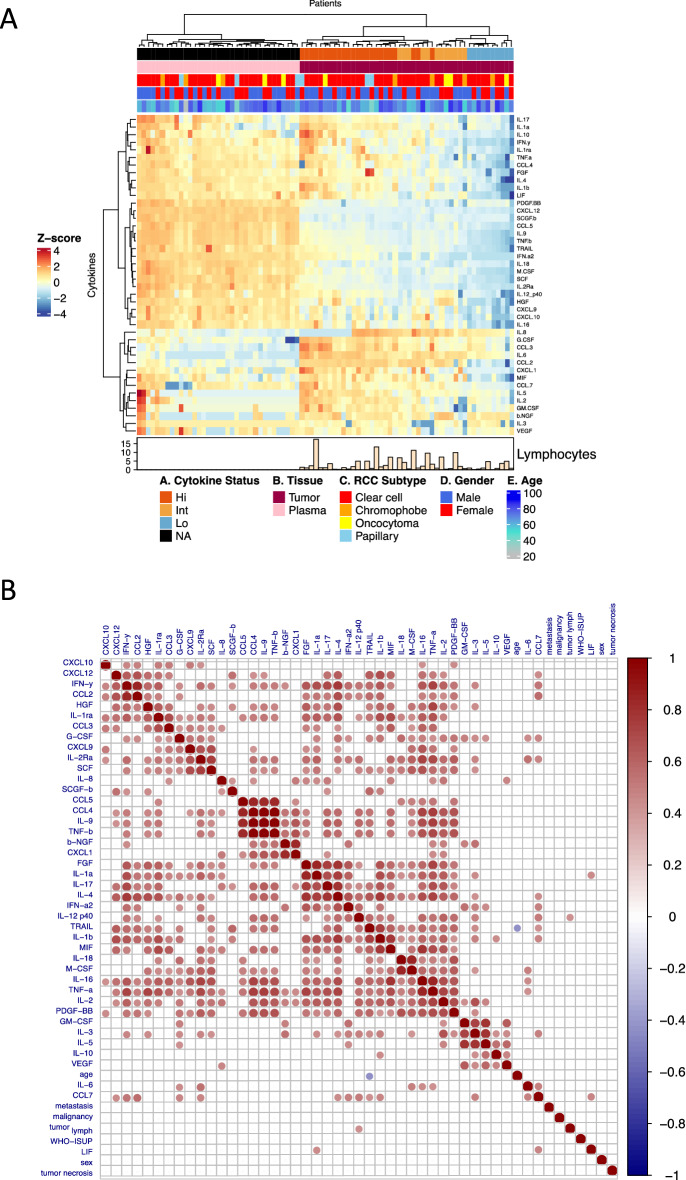
(**A**) Heatmap showing the circulating cytokine expressions from 33 matching plasma samples together with all tumor samples (n = 46). Unsupervised clustering showed markedly distinct clustering between the cytokine profiles of the different sample types, but no differences were observed with the clinical parameters. (**B**) Correlation plot of the plasma cytokines and various tumor clinical parameters such as the WHO/ISUP 2016 tumor grading, metastasis development, tumor malignancy, abundance of tumor lymphocytes, presence of necrosis in the tumor tissue, age, and gender of the patients. The quantity of the tumor lymphocytes was positively correlated with plasma IL-12 (p40) expression (Supplementary Fig. S6D). The full list of clinical parameters is found in Supplementary Table S1.

To identify whether peripheral blood cytokine profiles are able to predict the cytokine milieu in the tumor microenvironment, we measured the circulating cytokines from 33 matching plasma samples from our tumor cohort. Unsupervised clustering analysis revealed marked differences between the plasma and tumor cytokine profiles (Fig. 4A). Approximately one third of the cytokines were especially prevalent in the tumors (IL-8, G-CSF, CCL3 (MIP-1α), IL-6, CCL2 (MCP-1), CXCL1 (GRO-α), MIF, CCL7 (MCP-3), IL-5, IL-2, GM-CSF, β-NGF, IL-3 and VEGF); another third in the plasma samples (PDGF-BB, CXCL12 (SDF-1α), SCGF-β, CCL5 (RANTES), Il-9, TNF-β, TRAIL, IFN-α2, IL-18, M-CSF, SCF, IL-2RA, IL-12, HGF, CXCL9 (MIG), CXCL10 (IP-10), IL-16); and the rest of the cytokines (IL-17, IL-1α, IL-10, IFN-γ, IL-1RA, TNF-α, CCL4 (MIP-1β), FGF, IL-4, IL-1β, and LIF) did not show marked differences between the sample types. When we included the healthy tissue samples to the analysis, the plasma cytokines still clustered apart, whereas the tumor and healthy tissue samples clustered together (Supplementary Fig. S6A). Additionally, when the analysis was repeated with only the ccRCC cases, the plasma samples clustered separately from the tumor (Supplementary Fig. S6B) and healthy cases (Supplementary Fig. S6C).

Plasma cytokine levels were not correlated with the clinical parameters, such as WHO/ISUP 2016 tumor grading, tumor necrosis, metastasis, and sex (Fig. 4B). Only plasma TRAIL levels and age were negatively correlated, whereas a positive correlation with the plasma IL-12 (p40) and tumor lymphocytes was observed (Supplementary Fig. S6Di, ii, respectively). Furthermore, when we compared the levels of individual cytokines between the tumor and plasma samples, no correlations were found, suggesting that the plasma cytokines do not mirror the tumor cytokine environment (Supplementary Figs. S6E and S7Ai,ii). As tumor CXCL10 and CXCL9 concentrations were correlated with the quantity of intratumoral lymphocyte and CD3 + T cells, we wanted to confirm whether the plasma levels of these cytokines would give some guidance to the tumor lymphocyte counts. However, no correlations were found for both cytokines with the quantity of tumor lymphocytes and CD3 + T cells (Supplementary Fig. S7Bi–iv).

## Discussion

In this study, we measured 42 cytokines from RCC tumors together with the matching adjacent renal tissues and plasma samples, to investigate the cytokine profiles and correlate the findings with the immunophenotype of the TILs and clinical parameters. Our results show that the cytokine profiles found in the tumor are distinct from the healthy renal tissue and plasma counterparts. However, we observed that tumors with high cytokine expression signatures also have increased cytokine levels in the matching adjacent healthy tissues. Most of the cytokines showed significantly positive correlations with each other in their expression levels, suggesting that cytokine-enriched tumors have an overall inflamed tumor microenvironment. In addition, our tumor-plasma cytokine analysis revealed that the plasma cytokines poorly mirror those of the tumor environment. Although previous studies have analyzed the circulating cytokines in RCC^11,12,19^ and in other solid tumors^20–22^ from serum or plasma samples, our work describes the cytokine profile of the actual tumor microenvironment in relation to the circulating and matching adjacent healthy tissue.

While the proportion of the TILs did not significantly differ between the cytokine highly and lowly expressing tumors, CXCL10 (IP-10) and CXCL9 (MIG), known to belong to the CXC chemokine subfamily were associated with increased lymphocyte quantities in the tumor samples. The CXCL9-CXCR3 axis is mainly known to regulate cell migration activation and immune reactivity through the recruitment of activated T cells, NK cells and NKT cells^23^. Similarly, CXCL10 is released in response to IFN-γ, and is a chemotactic factor involved in the recruitment of activated T and NK cells to sites of inflammation^20^. Our results suggest that both cytokines are involved in the recruitment and homing of T and NK cells into the tumor microenvironment in RCC. However, as circulating CXCL10 and CXCL9 levels did not correlate with tumor lymphocyte counts, their analysis from the blood samples cannot be used as a biomarker for T-cell rich tumors, further highlighting the importance of analyzing cytokines in primary tumor samples.

Although most of the cytokines correlated with each other, IL-8 was a clear exception, and its levels did not correlate with any other cytokine. IL-8, also known as CXCL8, is a proinflammatory cytokine involved in the recruitment of immune cells and tumor progression via epithelial-to-mesenchymal transition^24^. Recent studies by Yuen et al. have shown that high baseline plasma IL-8 or on-treatment increased IL-8 levels correlated with an overall decreased survival and response rate to anti-PD-L1 therapy in patients with metastatic urothelial carcinoma and metastatic RCC^19^. Other studies have also shown that increased intratumoral IL-8 expression correlates with high levels of immune suppressive myeloid cells, such as neutrophils and monocytes^25,26^. Based on our data, the baseline tumor IL-8 expression does not correlate with metastasis formation, and the follow-up is too short to reveal whether the cytokine has an impact on the overall survival.

A caveat of our study is that our patient cohort did not receive any immuno-oncological treatments after their nephrectomy procedures, and the short follow-up limits correlating the findings with clinical parameters or treatment responses. Furthermore, our previous immunophenotyping analysis^13^ did not include markers for macrophages and other immune cells in addition to the lymphocytes. Tumors with a highly expressing cytokine profile thus most likely have increased quantities of other immune cells, such as those in the myeloid compartment that contribute to the immune inflamed tumor microenvironment. Therefore, further work including single cell analysis and functional studies are needed to provide a comprehensive understanding of cytokine signaling in the inflamed tumor microenvironment and the impact of individual cytokines in RCC.

## Supplementary Information

Supplementary Information 1.

Supplementary Information 2.

Supplementary Information 3.

## Data Availability

The datasets used and/or analyzed during the current study available from the corresponding author on reasonable request.
