# Supplementary material for: The tumor and plasma cytokine profiles of renal cell carcinoma patients
Source: Sci Rep. 2022 Aug 4;12:13416. doi: 10.1038/s41598-022-17592-3 (PMC9352752; doi:10.1038/s41598-022-17592-3)
Supplement: Supplementary file 1 — Supplementary Information 1. [file 41598_2022_17592_MOESM1_ESM.pdf]

Fig. S1

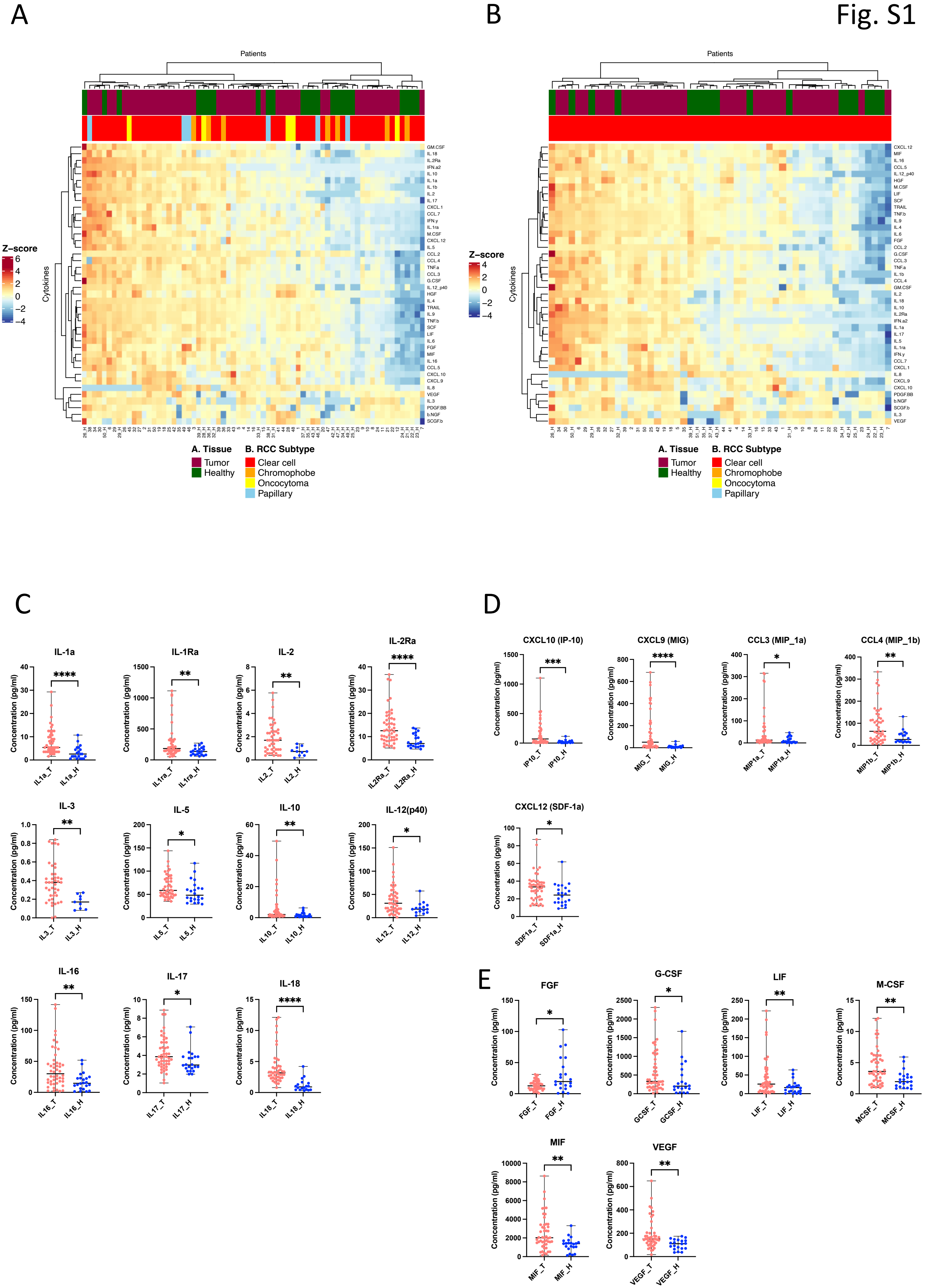

Availability of data and materials

The datasets used and/or analyzed during the current study available from the corresponding author on reasonable request.

Fig. S2

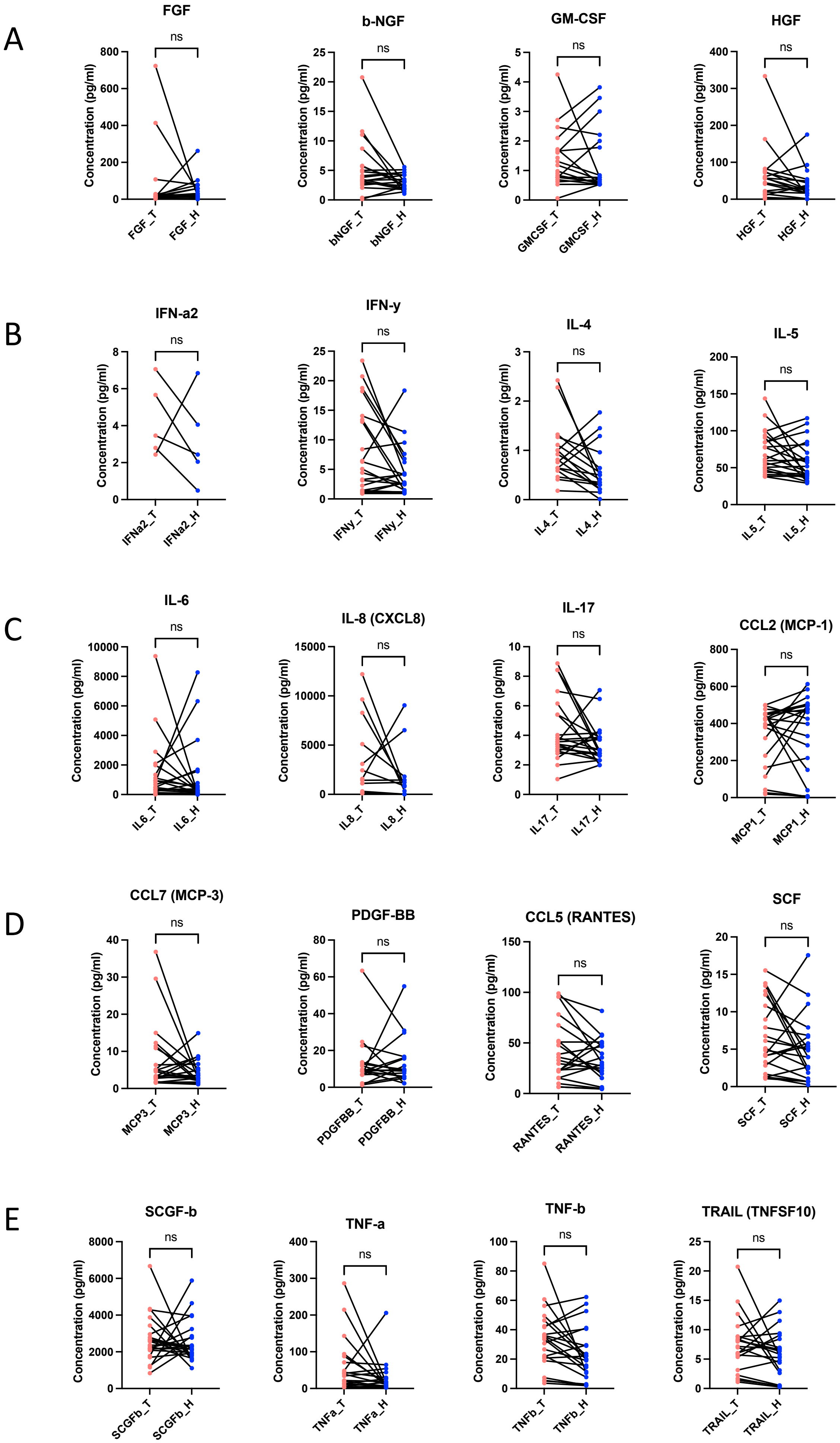
A
FGF
b-NGF
GM-CSF
HGF
ns
Concentration (pg/ml)
FGF_T
FGF_H
bNGF_T
bNGF_H
GMCSF_T
GMCSF_H
HGF_T
HGF_H
B
IFN-a2
IFN-y
IL-4
IL-5
IFNa2_T
IFNa2_H
IFNy_T
IFNy_H
IL4_T
IL4_H
IL5_T
IL5_H
C
IL-6
IL-8 (CXCL8)
IL-17
CCL2 (MCP-1)
IL6_T
IL6_H
IL8_T
IL8_H
IL17_T
IL17_H
MCP1_T
MCP1_H
D
CCL7 (MCP-3)
PDGF-BB
CCL5 (RANTES)
SCF
MCP3_T
MCP3_H
PDGFBB_T
PDGFBB_H
RANTES_T
RANTES_H
SCF_T
SCF_H
E
SCGF-b
TNF-a
TNF-b
TRAIL (TNFSF10)
SCGFb_T
SCGFb_H
TNFa_T
TNFa_H
TNFb_T
TNFb_H
TRAIL_T
TRAIL_H

Fig. S3

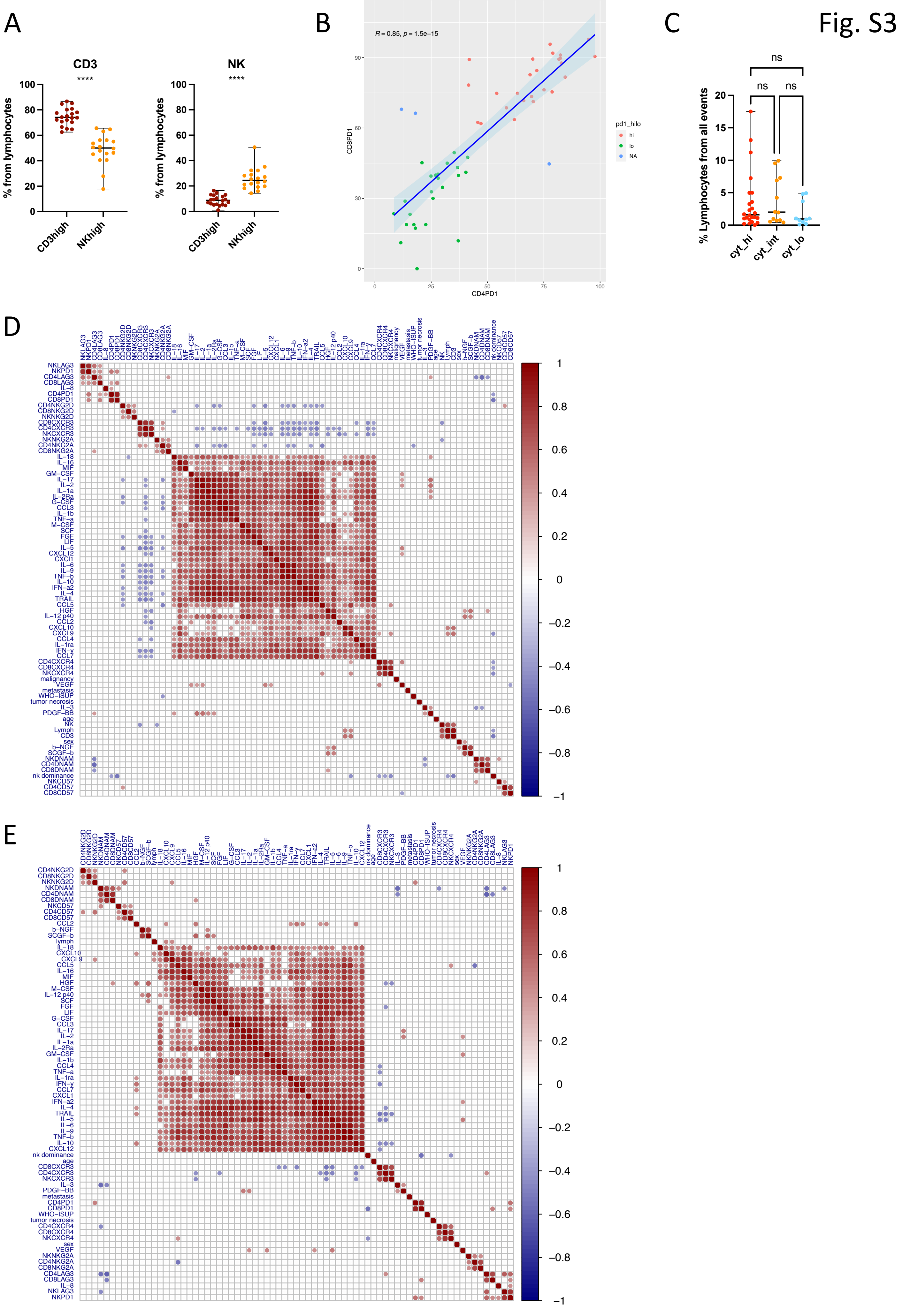

A
CD3
****
NK
****
% from lymphocytes
CD3high
NKhigh
B
R = 0.85, p = 1.5e−15
CD8PD1
CD4PD1
pd1_hilo
hi
lo
NA
C
ns
ns
ns
% Lymphocytes from all events
cyt_hi
cyt_int
cyt_lo
D
E

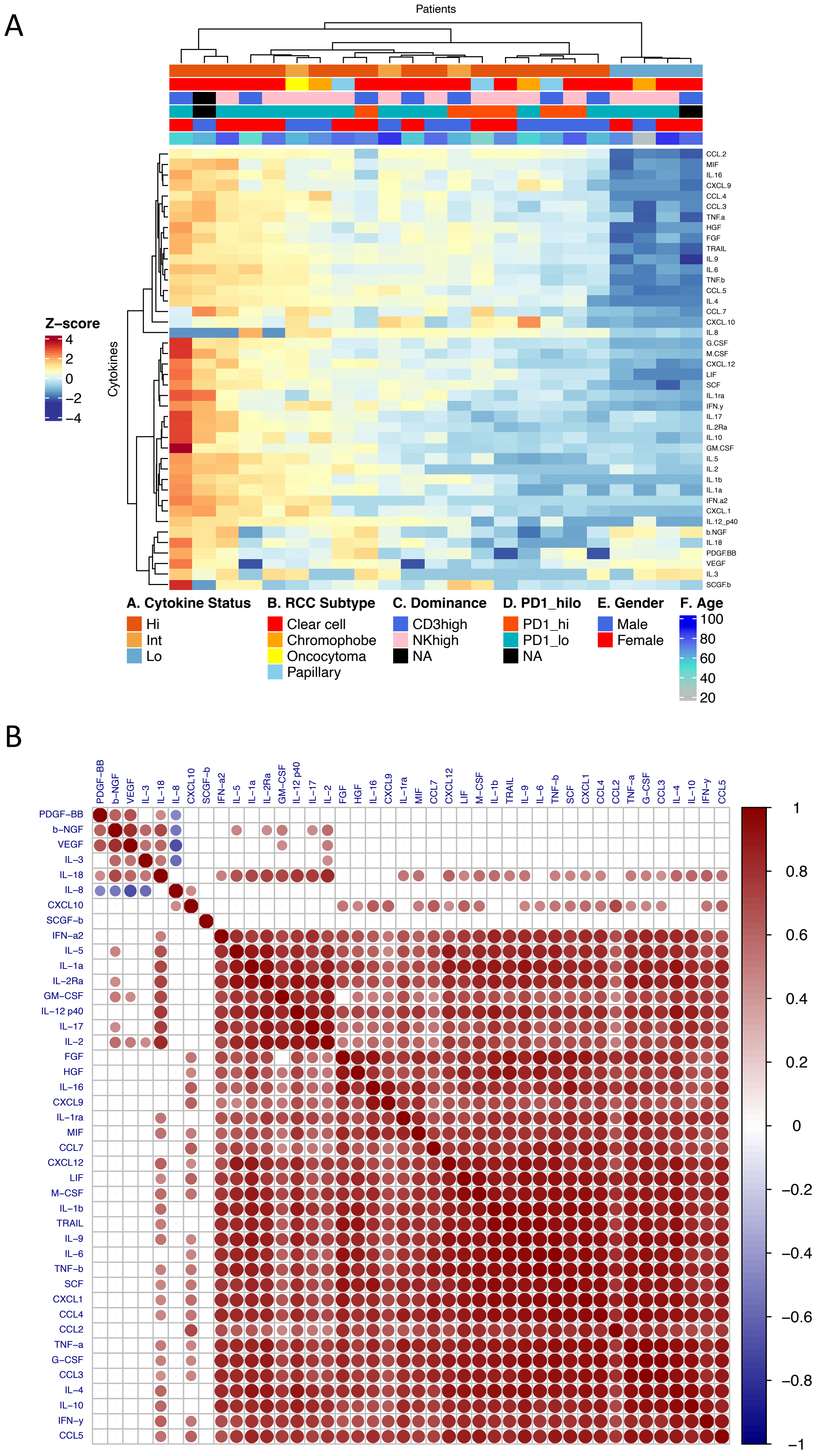
A
Patients
Z-score
4
2
0
−2
−4
Cytokines
CCL.2
MIF
IL.16
CXCL.9
CCL.4
CCL.3
TNF.a
HGF
FGF
TRAIL
IL.9
IL.6
TNF.b
CCL.5
IL.4
CCL.7
CXCL.10
IL.8
G.CSF
M.CSF
CXCL.12
LIF
SCF
IL.1ra
IFN.y
IL.17
IL.2Ra
IL.10
GM.CSF
IL.5
IL.2
IL.1b
IL.1a
IFN.a2
CXCL.1
IL.12_p40
b.NGF
IL.18
PDGF.BB
VEGF
IL.3
SCGF.b
A. Cytokine Status
Hi
Int
Lo
B. RCC Subtype
Clear cell
Chromophobe
Oncocytoma
Papillary
C. Dominance
CD3high
NKhigh
NA
D. PD1_hilo
PD1_hi
PD1_lo
NA
E. Gender
Male
Female
F. Age
100
80
60
40
20
B
PDGF−BB
b−NGF
VEGF
IL−3
IL−18
IL−8
CXCL10
SCGF−b
IFN−a2
IL−5
IL−1a
IL−2Ra
GM−CSF
IL−12 p40
IL−17
IL−2
FGF
HGF
IL−16
CXCL9
IL−1ra
MIF
CCL7
CXCL12
LIF
M−CSF
IL−1b
TRAIL
IL−9
IL−6
TNF−b
SCF
CXCL1
CCL4
CCL2
TNF−a
G−CSF
CCL3
IL−4
IL−10
IFN−y
CCL5
1
0.8
0.6
0.4
0.2
0
−0.2
−0.4
−0.6
−0.8
−1

Fig. S5

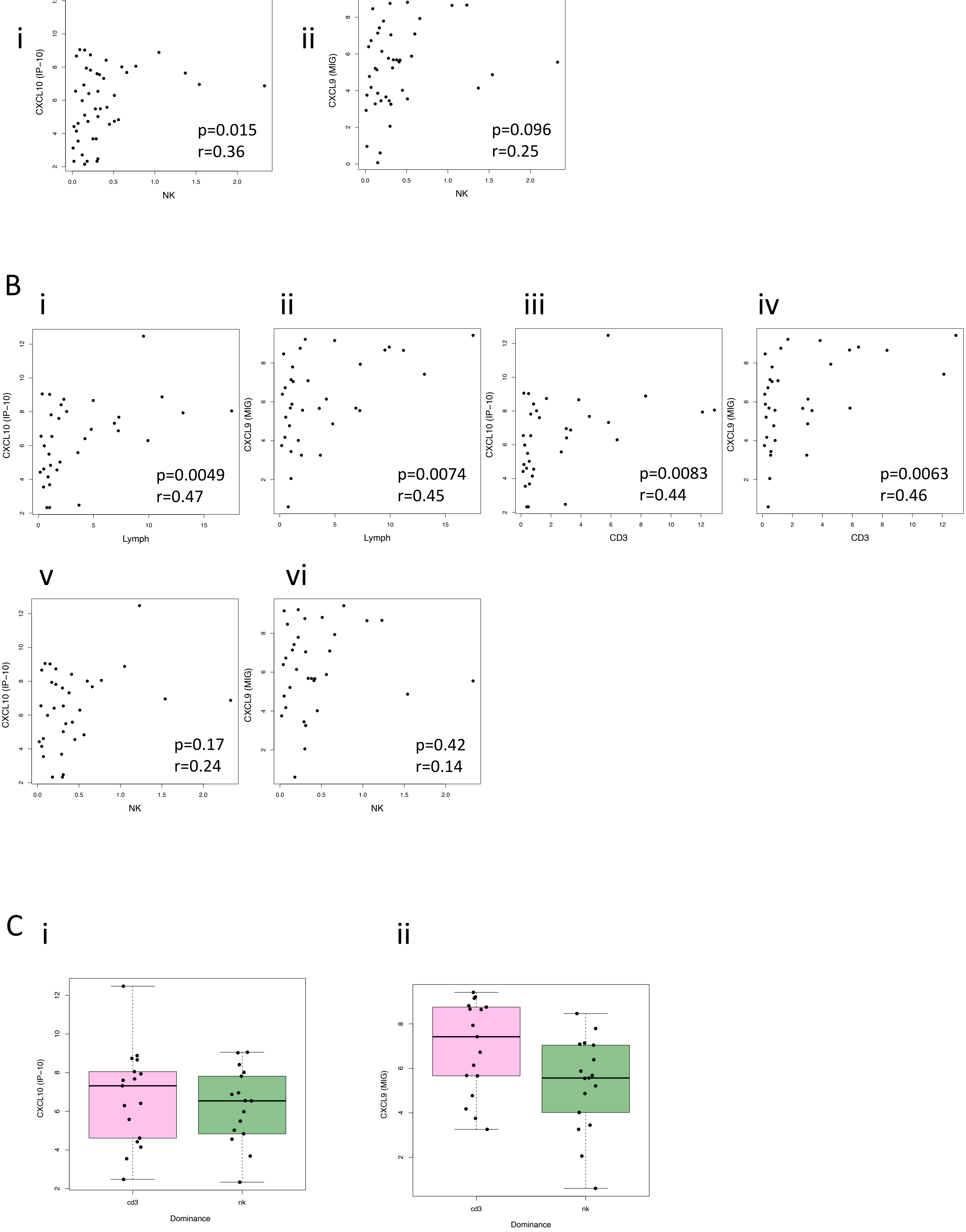
A
i
CXCL10 (IP−10)
NK
p=0.015
r=0.36
ii
CXCL9 (MIG)
NK
p=0.096
r=0.25
B
i
CXCL10 (IP−10)
Lymph
p=0.0049
r=0.47
ii
CXCL9 (MIG)
Lymph
p=0.0074
r=0.45
iii
CXCL10 (IP−10)
CD3
p=0.0083
r=0.44
iv
CXCL9 (MIG)
CD3
p=0.0063
r=0.46
v
CXCL10 (IP−10)
NK
p=0.17
r=0.24
vi
CXCL9 (MIG)
NK
p=0.42
r=0.14
C
i
CXCL10 (IP−10)
cd3
nk
Dominance
ii
CXCL9 (MIG)
cd3
nk
Dominance

Fig. S6

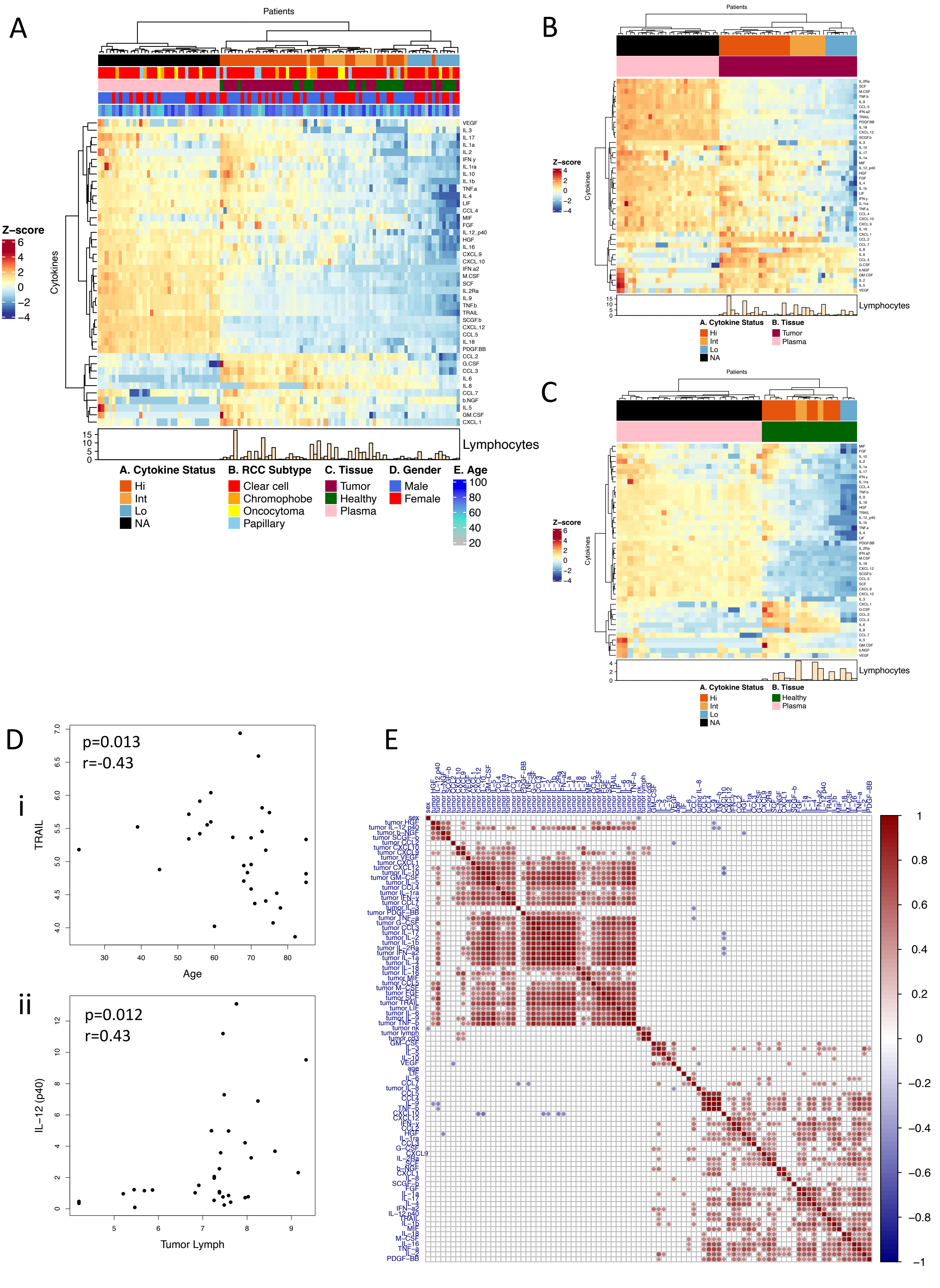

A
Patients
Z–score
6
4
2
0
−2
−4
Cytokines
VEGF
IL.3
IL.17
IL.1a
IL.2
IFN.y
IL.1ra
IL.10
IL.1b
TNF.a
IL.4
LIF
CCL.4
MIF
FGF
IL.12_p40
HGF
IL.16
CXCL.9
CXCL.10
IFN.a2
M.CSF
SCF
IL.2Ra
IL.9
TNF.b
TRAIL
SCGF.b
CXCL.12
CCL.5
IL.18
PDGF.BB
CCL.2
G.CSF
CCL.3
IL.6
IL.8
CCL.7
b.NGF
IL.5
GM.CSF
CXCL.1
Lymphocytes
A. Cytokine Status
Hi
Int
Lo
NA
B. RCC Subtype
Clear cell
Chromophobe
Oncocytoma
Papillary
C. Tissue
Tumor
Healthy
Plasma
D. Gender
Male
Female
E. Age
100
80
60
40
20
B
Patients
Z–score
Cytokines
Lymphocytes
A. Cytokine Status
Hi
Int
Lo
NA
B. Tissue
Tumor
Plasma
C
Patients
Z–score
Cytokines
Lymphocytes
A. Cytokine Status
Hi
Int
Lo
NA
B. Tissue
Healthy
Plasma
D
i
p=0.013
r=-0.43
TRAIL
Age
ii
p=0.012
r=0.43
IL−12 (p40)
Tumor Lymph
E

Fig. S7

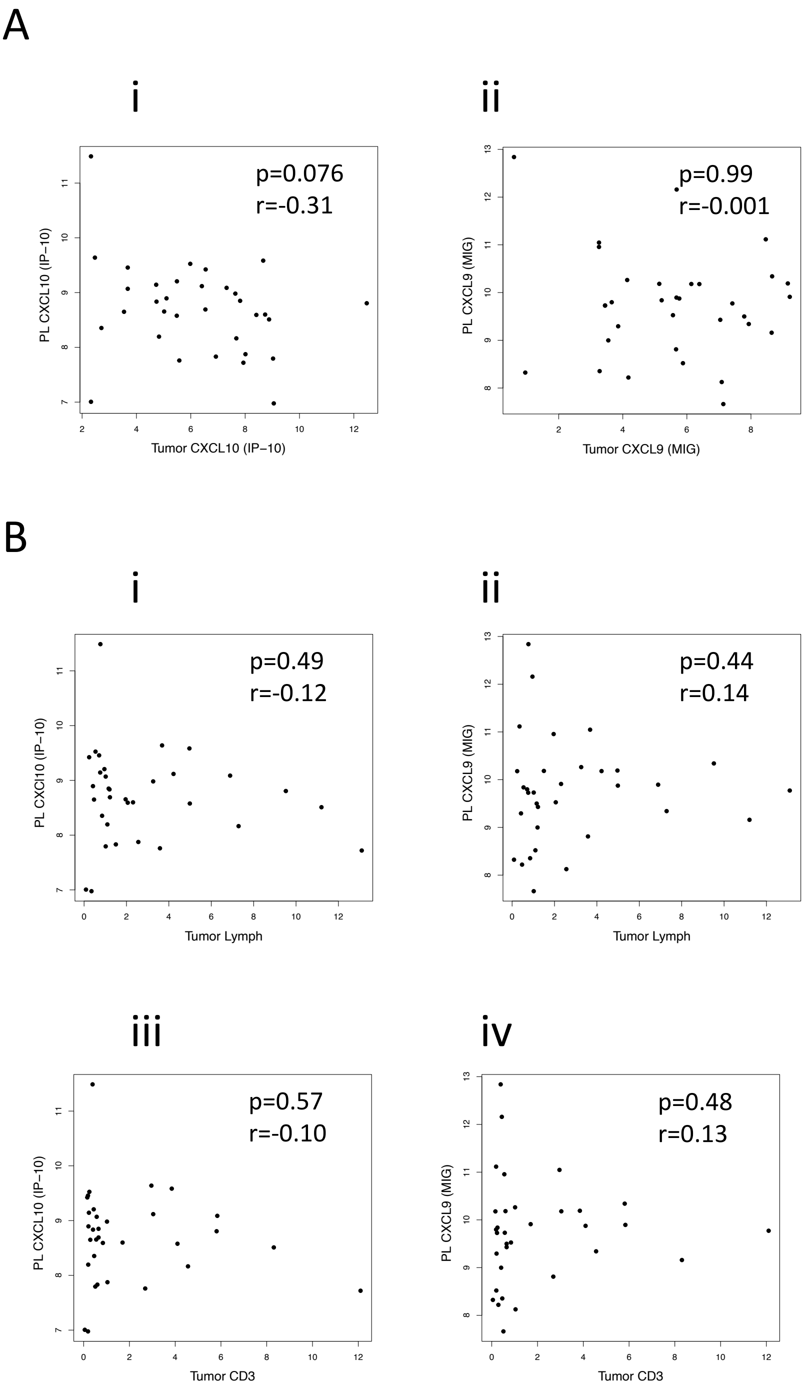
A
i
p=0.076
r=-0.31
PL CXCL10 (IP-10)
Tumor CXCL10 (IP-10)
ii
p=0.99
r=-0.001
PL CXCL9 (MIG)
Tumor CXCL9 (MIG)
B
i
p=0.49
r=-0.12
PL CXCl10 (IP-10)
Tumor Lymph
ii
p=0.44
r=0.14
PL CXCL9 (MIG)
Tumor Lymph
iii
p=0.57
r=-0.10
PL CXCL10 (IP-10)
Tumor CD3
iv
p=0.48
r=0.13
PL CXCL9 (MIG)
Tumor CD3
