# Supplementary material for: The tumor and plasma cytokine profiles of renal cell carcinoma patients
Source: Sci Rep. 2022 Aug 4;12:13416. doi: 10.1038/s41598-022-17592-3 (PMC9352752; doi:10.1038/s41598-022-17592-3)
Supplement: Supplementary file 2 — Supplementary Information 2. [file 41598_2022_17592_MOESM2_ESM.docx]

**Availability of data and materials**

The datasets used and/or analyzed during the current study available from the corresponding author on reasonable request.

**Supplemental figure legends**

**Table S1**

Table showing RCC patient data including clinical parameters and status

**Figure S1**

1. Heatmap of all tumor cases (n=46) and matching healthy tissue samples (n=23) shows two distinct clusters of highly and lowly expressing cytokines.
2. Heatmap of clear cell RCC cases (n=35) and their matching healthy tissue samples (n=17) shows similar highly and lowly expressing cytokine clusters. For all heatmap analyses, unsupervised Euclidean distance clustering and ward.D2 linkage methods were used

C.-E. Dotplots showing differences in cytokine profiles between all tumor (_T) and adjacent healthy renal tissue (_H) samples (matched samples are shown in Figure 1). Overall, tumors displayed higher levels of most of the cytokines compared to the healthy counterparts. Non-parametric Mann-Whitney U test was used with a 95% confidence level. Error bars indicate the median and range. For all graphs: ns, not significant; *, p<0.05; **, p<0.01; ***, p<0.001; ****, p<0.0001.

**Figure S2**

A.-E. Pairwise comparisons of each cytokine between the tumor (_T) and matching healthy tissue (_H) samples. Wilcoxon matched-pairs signed rank statistical testing with a 95% confidence level was used. ns=non-significant.

**Figure S3**

1. Dotplots showing that CD3+ T cell dominant (CD3high) tumor samples have an increased abundance of CD3+ T cells than NK cell dominant (NKhigh) tumors, and that NK cell dominant tumors are more enriched with NK cells compared to the CD3high counterparts. Non-parametric Mann-Whitney U test was used with a 95% confidence level. Error bars indicate the median and range. ****, p<0.0001.
2. Scatterplot showing two subgroups (PD1_hi (pink) and PD1_lo (green)) based on the CD4+ T cell PD-1 and CD8+ T cell PD-1 expressions. Blue dots (NA) indicate samples that have been excluded from the analysis.
3. Scatterplot showing Kruskal-Wallis test comparing the absolute amount of lymphocytes from all events and the different cytokine clusters (hi, int, lo). Benjamini-Hochberg multiple correction with a family-wise alpha threshold and confidence level of 0.05 were used. cyt_hi = high, cyt_int = intermediate, cyt_lo = low, ns=non-significant.
4. Correlation plot showing the cytokine profile of all tumors (n=46) together with various clinical parameters including the quantity of lymphocytes, CD3+ T and NK cells out of all cells in the tumor. For all correlation plots, Spearman rank correlation across the samples was used and Benjamini-Hochberg corrected p-values with a false discovery rate (FDR) < 0.05 were considered significant.
5. Correlation plot showing the cytokine profile in clear cell RCC (ccRCC) cases (n=35) with clinical parameters.

**Figure S4**

1. Heatmap of the adjacent healthy renal tissue samples (n=23) expressing a total of 42 cytokines, including the clinical parameters such as gender, and RCC subtype. Unsupervised Euclidean distance clustering and ward.D2 linkage methods were used. Similar to the RCC tumors shown in Fig. 2A, the healthy adjacent tissue samples were grouped using three main clusters according to the matching high (hi), intermediate (int) and low (lo) tumor cytokine expression profiles. No differences were observed between the clinical parameters. NAs refer to samples with missing clinical data.
2. Correlation plot of the healthy adjacent renal tissue cytokine profiles. Strong positive correlations were observed across most of the healthy sample cytokine expressions. Although IL-8 did not show any correlation in the tumor samples, negative correlations were observed between IL-8 and a few cytokines (PDGF-BB, β-NGF, VEGF and IL-3).

**Figure S5**

1. Scatterplots showing Spearman’s correlation between the expression of cytokines (i) CXCL10 (IP-10) and (ii) CXCL9 (MIG) with tumor NK cell abundance. p=p-value, r=Spearman’s rho.
2. Scatterplots showing Spearman’s correlation between (i) CXCL10 and (ii) CXCL9 with the quantity of tumor lymphocytes from the ccRCC cases alone. Similar observations were made between (iii) CXCL10 and (iv) CXCL9 with the CD3+ T cell abundancy in the ccRCC cases, but there no correlations were observed between the two cytokines ((v) and (vi), respectively) and the quantity of NK cells in the ccRCC tumors.
3. Boxplots showing elevated expressions of (i) CXCL10 (p=0.56) and (ii) CXCL9 (p=0.03) in the CD3+ T cell dominant tumors compared to the NK cell dominant ccRCC cases.

**Figure S6**

1. Heatmap of all the tumor, matching adjacent healthy kidney and plasma sample cytokines, showing distinct clustering between the plasma and tumor-healthy samples.
2. Heatmap showing distinct differences between cytokines in the tumor (n=35) and matching plasma samples (n=26) in just the ccRCC cases.
3. Heatmap showing that the matching healthy adjacent kidney samples (n=17) and plasma (n=26) samples from only ccRCC cases show distinct cytokine expression patterns.
4. Positive Spearman’s correlation revealed (i) negative correlation with plasma TRAIL levels and age of the patients, but positive correlation with plasma IL-12 (p40) levels and the quantity of tumor lymphocytes (ii).
5. Correlation plot showing the cytokine profiles between the tumor and plasma samples including the clinical parameters. Most of the cytokines from the tumor clearly form a distinct profile apart from the plasma cytokines. Few negative correlations were observed between the intratumoral and circulating cytokines.

**Figure S7**

1. ­Scatterplots showing Spearman’s correlation between (i) CXCL10 and (ii) CXCL9 expression levels in the plasma compared to the tumor. No correlations were observed, confirming that the tumor and plasma cytokine profiles are distinct from each other.
2. Plasma (PL) CXCL10 and CXCL9 expressions do not correlate with the quantity of tumor infiltrating lymphocytes (i and ii, respectively), nor with the CD3+ T cell abundance in the tumor (iii and iv, respectively).
